# Metallic Crowns

**Published:** 1883-05

**Authors:** William N. Morrison


ARTICLE II.

### METALLIC CROWNS.

BY WM. N. MORRISON, D. D. S., OF ST. LOUIS, MO.

About fourteen years ago, I first performed the operation of adjusting gold crowns to crownless teeth, and gave it to the profession, and through them to the public, under the head of

A NEW OPERATION.

[Page 184, Missouri Dental Journal, 1869.]

Miss W ——— came to me with a first left lower molar decayed to the extent that the entire lingual and a greater part of the labial surfaces below the gum were removed. The roots were filled properly with gold, and the crown had been filled several times ; the last time the crown was built up in good style, but the two walls being of such a shape that the filling could not be made self-retaining, I took a natural tooth, corresponding as nearly as possible in size and shape to its fellow of the opposite side, and imbedded its roots in plaster, to make a model from which to get a metallic die, over which to swage a gold cap.

" I used a piece of thin gold plate, cutting it at the corners, giving but slight lap for soldering after it was perfectly fitted to the die. I then fitted this cap accurately to the remaining portion of the tooth in the patient's mouth, allowing it to extend under the free margin of the gum, quite to the alveolus, which was about the thirty-second of an inch below the margin of decay.

"After soldering a bar across the cap, from the lingual to the labial surfaces, it was finished at the lathe. I then prepared the patient's mouth as usual for filling; made a thin paste of oxy-chloride of zinc; filled the cap, and pressed it to its place; the superfluous cement was crowded out of the cap and removed at the margin of the gum.

"I had the pleasure of seeing that tooth to-day, nearly four months after the operation, and had the gratification of seeing and hearing it pronounced a perfect success. N."

Since that time but very few have availed themselves of the advantages of this, *the grandest invention in operative dentistry during the last quarter of a century.*

Take, for example, any case where extensive building up is required. A large amount of watchful care, physical outlay and skill on the part of the operator (acquired only by the few) in conjunction with the most approved, rapid and painless methods at our command for welding the improved forms of gold, is necessary to produce the most characteristic and finished result; and this only after a five or six hours' seige over a patient, who has but a small stock of endurance to start with, and in consequence, has grown more restless and irritable each hour; the question is forced upon us, "Did it pay?" Then, when tested by the tooth of time, the thermal changes acting upon the large metallic mass, the walls show checks, stains and cracks, and finally fall away, leaving, unsupported, the large over-malleted nugget, then we are forced to say with the patient, who refuses to submit to the inquisition again, "It did not pay."

"Too far gone to be filled again, have it out." And as the dentist (O. S.) dances around showing the large well-formed roots in the forceps, the triumph of his skill, he lays the flattering unction to his soul that "dead men tell no tales."

We will all agree that large masses of metal are not the correct thing to be worn in teeth with live pulps at any time, though we are continually trying the experiment to see under how much gold a pulp will drag out a miserable existence. The crowning method comes grandly to our relief in all such cases. The pulps can be saved alive and the tooth made more comfortable and durable than by any other method yet known.

I have been operating upon an entirely different basis for ten years and more. Teeth, or roots, so loose that they can be removed with the thumb and finger, I do extract, when I feel they are beyond the hope of successful treatment, but no others.

I hope the day is not far distant when the forceps, extracting screws, gas, and every facilitating medium by which teeth and sound roots are extracted, will be forever laid aside, labeled machines of torture of the past, to be used like the turnkey, only in rare cases and at long intervals, and when there will be enacted laws which will make it punishable by fine and imprisionment for the extraction of teeth or roots that are firm in the jaws, or are susceptible of being made so. A patient suffering from pressure upon an exposed pulp in a tooth is a correct judge of what should be done with it, and a dentist who accedes to the demand to extract, should be held accountable before the law for mayhem—even more than for the mutilation of any other part of the anatomy, for upon the proper mastication of food all the other parts of anatomy depend.

I have had cases, pronounced by other operators hopeless, useless and threatening harm, where I have adjusted crowns which have been worn with great profit to the patient for years; several after unsuccessful attempts at extraction where the roots had been fractured uncomfortably high up under the gum and of inconvenient shape ; where the restorations have been pronounced marvelous, and they really were satisfactory beyond anything that was expected of them.

Another class of patients for whom this style of operating if particularly desirable, is where chemical abrasionis accompanied by extreme sensitiveness of the dentine, and a nervous dread of instruments; and chemico-mechanical abrasion where it is extensive, and formidable restorations required—illustrated in case No. 1. Mr. O. W., a gentleman about fifty-three years of age, retired merchant, nervous temperament, who in his early years was so occupied in business that he never took any care of his teeth, but had them extracted as soon as they troubled him, resulting in the loss of the molar teeth in lower, and molar and both second bicuspids in the superior jaw. The crowns of the remaining teeth were so extensively abraded that the lower incisors were occluding into the upper gums, and the upper bicuspids were occluding into the lower gum, pulps alive in all but three. As these teeth had been doing double duty for years, it was necessary to reconstruct them of such material, shape and proportions as would best fit them for mastication, as well as incisive and enunciative functions.

The six superior stumps were fitted with truncated cone shells of twenty-two caret gold, thickness twenty-eight guage plate, the small ends fitted tightly to the roots, and the remaining portion planished over a round, pointed stake into proper anatomical shapes and adjusted to each other and trimmed to the margin of the lips, using the surplus material on the palatine side to increase the masticating face as much as possible. The coronal faces were closed with heavy platinum gold, slightly sunken below the edge, and well thickened with solder on the inside. The bicuspids were made without taper, but slightly closed in at cervical margin, to take a firm grasp upon the roots just at the free margin of the gum. Upon the coronal faces of heavy gold were soldered cresent-shaped clippings for cusps to facilitate mastication. The lower bicuspids were made similar to upper; the lower canines were capped with truncated cones with their bases fitted to the tooth about an eighth of an inch from the gum. They were put on in pairs at each sitting with phosphate cement, a small vent-hole being required in several. In the three canals where the pulps were dead, heavy retaining screws were used; the other stumps were not cut or roughened but merely cleansed with chloroform and well dried. The uneven edges of the lower incisors were leveled with platinum gold foil. From the patient's experience with them, he said that he and the dentist might live to make a restoration equal to *one* of the other crowns, but they never would live through a *second*.

Mr. J. W——, a gentleman about forty years of age, prominent business man, identified with large capitaled interests, who has for years chewed cigars which are made of the outside sandy leaves of the plant, instead of prepared or medicated tobacco, called chewing tobacco. Ten years ago the upper teeth were worn through the enamel, and so much cupped out that I was obliged to make quite extensive restorations with retaining screws and cohesive gold, all of which remain in good condition to-day, though considerably worn.

The abrasion was only on the right side; by no motion of the jaw could he get the teeth within an eighth of an inch of each other; the lower ones were cupped out until the enamel margins stood sharp—dentine very sensitive; he had deferred consulting me for want of time to devote to it, as he remembered the sittings for former work. He asked me to devise some means of relief from the stretching of his mouth and the long sittings, all of which was accomplished by using thin ribbons of platinum for the bands, so as not to increase the size of the crowns; the coronal faces were made of heavy platinum gold, shod with four half cresents, as described above. With thin phosphate cement, the force of the closing of the jaw was all that was required to make the work complete. I left the empty crowns in position until the cemented ones were secured, and then cemented them.

After the barrel of the crown of 24 carat gold has been fitted to the root and planished into correct anatomical shape, shorter than the finished crown by a sixteenth or eigth of an inch, the swaged coronal end of thin platinum (30 guage plate) is slightly stuck to it with solder, 22 carat gold in two or three places, when it is placed upon the plaster model, or in the mouth, and correctly articulated, the thin metal readily yielding to occluding cusps. The lap neatly planished and soldered, thickening the occluding face upon the inside to a sixteenth of an inch or more as required, with little clippings of platinum and plate gold (20 carat) for solder. Where platinum is used for the whole shell of the crown, but little care is required in soldering and filling with 24 carat gold.

Any face of these dies may also be used in swaging metallic sections to protect cement fillings, leaving uncovered those portions of enamel in a healthy condition. The sections should overlap the margins about the thirty-second of an inch, be burnished carefully to them and should have anchorage soldered to its inner surface to secure it to the cement.

In shape and distribution of material, making them thin at the cervical margins and thick at the cusps, I endeavor to reproduce in metal a copy of nature's enamel.

DISCUSSION.

Dr. Brophy explained the necessity of trimming the exposed ends of incisor roots so that their sides shall be parallel, allowing the upper margin of the band to fit close after being driven on. He described (with the help of a drawing on the blackboard) how to make secure a leaning under tooth where the leverage is very strong for the tipping over of the crown. The band is carried very low down on the side needing most support, and a loop of metal being soldered to the under side of the masticating surface, extending downward into the pulp chamber, which is well undercut, the whole fastened in place with cement, makes a very secure operation. Uses coin gold instead of pure gold.

Dr. A. E. Matteson : I have been very much interested in Dr. Morrison's description, and in his articles in the *Missouri Dental Journal* upon this subject.

I think it is important to retain the portion of enamel or cementum at the lower end of the upper incisor roots, which Dr. Brophy has just advocated the removal of.

I make crowns of pure gold and platina plate, which is made by sweating pure gold on platina and rolling to No. 35 plate guage. This can be burnished over metal "forms."

I make these "forms" by taking an impression of a natural tooth, similar to the one I wish to reproduce, using a putty made of glycerine and litharge, in two or more sections. This putty made as stiff as it can be worked, dries rapidly when subjected to a gentle heat.

After removing the impression, wire together and fill with Babbitt metal. Over this form I make a pattern, using "Stuck's polishing tin," cutting out where it laps over the grinding surface, and also cutting out an opening, such as a simple crown cavity would present, remove and flatten without stretching the margins.

Having taken a measurement of the circumference of the root at the margin of the gum with fine binding wire, I cut the gold and platina plate to the tin pattern, making it longer or shorter as the measurement of the circumference of the root may be. Then burnish the plate over the form, remove and solder the ring thus formed as well as the cuts on the grinding surface, using 20 carat solder ; try on the root, and it should fit snugly and up nearly to the alveolar process. If the articulation is not correct mark where it strikes and with a V shaped file cut out, press the edges together, and solder ; repeat until articulation *is* perfect, when it is ready to anchor.

I use for his purpose one or more platina screws, inserted deep in the nerve canal, with the outer ends extending two-thirds the length of the crown. The screws should take hold firmly, and if more than one, stand at different angles in the crown.

If but one screw is used the nerve canal should be enlarged one-third its length with a cone-shaped burr, with its base towards the apex and an amalgam filling thoroughly packed around the screw in this cavity.

[TO BE CONTINUED.]

